# Extra-anatomic aortic bypass for repair of type A interrupted aortic arch associated with multiple aneurysms of the collateral circulation

**DOI:** 10.1186/1749-8090-8-S1-P39

**Published:** 2013-09-11

**Authors:** NAG Stolf, MLP Sobral, A Terrazas, E Pires, P Britto, GG Santos

**Affiliations:** 1 Unidade de Doenças Torácicas Stolf, Hospital Beneficência Portuguesa de São Paulo, São Paulo, Brazil

## Background

Interrupted aortic arch in adults (18 years and older) is a rare condition which medical literature reports only few cases of isolated anomaly. If it is left untreated, 90% of the affected infants die at a median age of 4 days.

## Methods

A 22 years old Asian woman with a history of cooling, swelling and pain of upper part of body during exercise, and cyanosis of left arm and both legs. At physical examination, she presented diminished pulses of the left arm and inferior limbs, and high blood pressure in the right arm. A Doppler showed switched flow in the left vertebral artery, and occlusion of left subclavian artery with a refilling through left vertebral and internal thoracic arteries. A thoracic CT scan diagnosed a type A interrupted aortic arch with very large aneurysms of collateral arteries. A single stage extra-anatomic procedure of ascending-to-descending thoracic aorta bypass grafting technique was performed through a median sternotomy and a posterior pericardial approach. Total occluding vascular clamps were used for the distal anastomosis of Dacron graft to the descending thoracic aorta. After the distal anastomosis, the left subclavian artery was ligated at its origin and the descending thoracic aorta, and proximally to the distal anastomosis, to prevent a rupture of the aneurysmatic collateral arteries.

## Results

The procedure had no complications and the inferior limbs were warm and left arm presented a satisfactory perfusion without symptoms. After three months, a thoracic CT scan showed excellent graft position and regression of the collateral arteries. After a year, the patient had no symptoms or complications.

## Conclusion

Our patient presents a type A interrupted aortic arch with collateral aneurysms treated by an extra-anatomic aortic bypass that is an efficient surgical strategy for this particular type of presentation.

